# Real-Time Artificial Intelligence for Early Sepsis Prediction Using Dynamic Clinical Data: A Systematic Review

**DOI:** 10.7759/cureus.113193

**Published:** 2026-07-22

**Authors:** Bhavna Singla, Priya Gupta, Anum Fatima, Shivam Singla, Sunita Kumawat, Vimi Bansal, Daniel E Cook, Taha Khalid

**Affiliations:** 1 Internal Medicine, Erie County Medical Center, Buffalo, USA; 2 Internal Medicine, Punjab Institute of Medical Sciences, Jalandhar, IND; 3 Internal Medicine, Fatima Jinnah Medical University, Lahore, PAK; 4 Internal Medicine, TidalHealth Peninsula Regional, Salisbury, USA; 5 Internal Medicine, St. Francis Medical Center, Lynwood, USA; 6 Internal Medicine, Maharishi Markandeshwar University, Bathinda, IND; 7 Internal Medicine, Avalon University School of Medicine, Willemstad, CUW; 8 Internal Medicine, Nishtar Medical University, Multan, PAK

**Keywords:** deep learning, early prediction, electronic health records, intensive care unit, machine learning, real-time monitoring, sepsis

## Abstract

Sepsis remains a major cause of morbidity and mortality worldwide, and delayed recognition continues to compromise timely intervention. In recent years, artificial intelligence (AI) has been increasingly applied to continuously updated clinical data to facilitate earlier detection of sepsis; however, the quality, interpretability, and clinical readiness of these models remain uncertain. This systematic review evaluated real-time AI models designed for early sepsis prediction using dynamic hospital data. A structured search of PubMed/MEDLINE, Scopus, and Web of Science identified studies published between January 2015 and June 2025. Eligible studies included adult hospitalized populations, employed machine learning or deep learning approaches using sequential or continuously updated data, and reported predictive performance for sepsis onset detection. Eight studies met the inclusion criteria. These studies were conducted across intensive care units, emergency departments, and multicenter hospital systems, with sample sizes ranging from several hundred to more than 500,000 patients. Model architectures included gradient boosting methods, neural networks, recurrent survival models, and deep learning prediction platforms. Reported discriminatory performance was moderate to high, with area under the receiver operating characteristic curve values generally ranging from 0.83 to above 0.95, and several studies demonstrated clinically meaningful lead times before sepsis onset or treatment initiation. More recent investigations increasingly incorporated external validation, transfer learning, false-alert mitigation strategies, and explainability methods, such as feature attribution and Shapley additive explanations analysis. Risk of bias assessment using the Prediction model Risk Of Bias ASsessment Tool indicated that most studies had a moderate overall risk of bias, primarily due to retrospective design, heterogeneous sepsis definitions, and limitations in analytical reporting. Current evidence suggests that real-time AI shows considerable promise for early sepsis recognition; however, prospective validation, calibration assessment, workflow integration, and demonstration of consistent patient benefit remain essential before widespread clinical implementation can be justified.

## Introduction and background

Sepsis is a time-sensitive syndrome characterized by a dysregulated host response to infection that can lead to life-threatening organ dysfunction, prolonged hospitalization, and substantial mortality. It remains a major challenge for health systems worldwide because early clinical manifestations are often nonspecific and may overlap with other forms of physiological deterioration [1]. Delayed recognition is associated with worse outcomes, whereas timely administration of antimicrobials, source control, hemodynamic support, and structured care bundles has been linked to improved survival in appropriately selected patients. Despite advances in critical care and the implementation of standardized management pathways, early identification of sepsis continues to be difficult in routine clinical practice, particularly in settings with high patient acuity, complex comorbidity profiles, and large volumes of continuously generated clinical data [2,3].

Conventional approaches to sepsis detection have relied on bedside assessment, laboratory trends, and rule-based screening tools such as the Systemic Inflammatory Response Syndrome (SIRS) criteria, the quick Sequential Organ Failure Assessment (qSOFA), the National Early Warning Score (NEWS), and institution-specific early warning systems [4,5]. Although these methods can support situational awareness, they are limited by modest specificity, variable sensitivity, delayed triggering, and reliance on static thresholds that do not adequately capture nonlinear and time-dependent patterns of physiological deterioration. In recent years, artificial intelligence (AI) and machine learning (ML) methods have been increasingly applied to electronic health records (EHRs), bedside monitoring streams, and longitudinal clinical data to enhance early recognition. These models are capable of integrating multiple variables simultaneously, updating risk estimates dynamically, and identifying subtle trajectories that may precede overt clinical decline [6]. However, improved statistical performance does not necessarily translate into clinical utility, and important concerns persist regarding false-alert burden, calibration, generalizability across institutions, fairness, and the interpretability of complex models.

The concept of explainable or interpretable AI has therefore gained increasing relevance in sepsis prediction research. Clinicians are more likely to engage with decision-support systems when the underlying basis of a prediction can be understood, contextualized, and translated into actionable clinical decisions. At the same time, many published sepsis prediction studies remain retrospective in design, employ heterogeneous outcome definitions, evaluate variable prediction windows, and report performance metrics inconsistently [7]. Consequently, although the evidence base is expanding rapidly, it remains difficult to synthesize in a manner that directly informs bedside implementation. A focused appraisal of real-time systems that utilize continuously updated clinical data may help identify the most promising methodological approaches and highlight critical gaps in the current literature [8].

The objective of this systematic review was to evaluate the performance, interpretability, and clinical relevance of real-time AI models designed for early sepsis prediction using continuously updated hospital data. Specifically, this review aimed to synthesize evidence regarding predictive accuracy, lead time before sepsis recognition, approaches to explainability, external validation, and factors influencing successful translation into routine clinical practice.

## Review

Methodology

Study Design and Reporting Framework

This study was conducted as a systematic review of published literature evaluating real-time AI models for early sepsis prediction using continuously updated hospital data. The review methodology was informed by the Preferred Reporting Items for Systematic Reviews and Meta-Analyses (PRISMA) framework to promote transparent identification, screening, eligibility assessment, and inclusion of relevant studies [9]. Given the emphasis on prediction model research, methodological considerations from the Prediction model Risk Of Bias ASsessment Tool (PROBAST) were also incorporated during critical appraisal of included studies [10]. The review question was structured according to a modified Population, Intervention, Comparison, Outcome (PICO) framework [11], in which the population comprised hospitalized adult patients at risk of sepsis; the intervention was real-time AI or ML-based prediction systems using dynamic clinical data; the comparator included conventional screening tools, standard care, or alternative predictive models where available; and the outcomes included predictive performance, prediction lead time, interpretability, external validation, and indicators of clinical utility.

Data Sources and Search Strategy

A structured electronic literature search was performed in PubMed/MEDLINE, Scopus, and Web of Science. These databases were selected to capture biomedical, clinical, and interdisciplinary AI literature. The search covered studies published from January 1, 2015, to June 30, 2025. This date range was chosen to reflect the modern period of ML-enabled sepsis prediction and to encompass the earliest foundational real-time studies identified in this review through the most recent external validation studies. Search terms were developed iteratively using controlled vocabulary and free-text keywords related to sepsis, prediction modeling, AI, and real-time monitoring. Representative search syntax included combinations of terms such as (“sepsis” OR “septic shock”) AND (“artificial intelligence” OR “machine learning” OR “deep learning” OR “neural network”) AND (“prediction” OR “early warning” OR “detection”) AND (“real-time” OR “continuous” OR “dynamic” OR “time series” OR “electronic health record” OR “ICU”). Boolean operators AND and OR were used to combine concepts, and database-specific indexing terms were applied where appropriate. Reference lists of eligible articles were also manually screened to identify additional relevant studies.

Study Selection and Eligibility Criteria

Titles and abstracts identified through the search strategy were initially screened for relevance, followed by full-text assessment of potentially eligible studies. Studies were included if they evaluated adult hospitalized populations in intensive care units (ICUs), emergency departments (EDs), or general inpatient settings; employed AI, ML, or deep learning (DL) methods to predict the onset of sepsis or septic shock; utilized continuously updated, sequential, or repeatedly sampled physiological, laboratory, or EHR data; and reported at least one measure of predictive performance, such as area under the receiver operating characteristic curve (AUROC), area under the precision-recall curve (AUPRC), sensitivity, specificity, false-alert burden, or prediction lead time. Studies incorporating external validation, multicenter cohorts, temporal validation, interpretability methods, or clear implementation relevance were considered particularly informative for the objectives of this review.

Studies were excluded if they focused exclusively on mortality prediction after sepsis diagnosis, involved pediatric populations or non-human subjects, or employed purely static admission-risk models without temporal updating. Editorials, narrative reviews, conference abstracts lacking sufficient methodological detail, and studies not directly related to sepsis onset prediction were also excluded. Reports centered solely on workflow implementation without primary model performance outcomes were considered supplementary but were not included as core evidence. These eligibility criteria were designed to ensure that included studies directly addressed the central research question of real-time early sepsis prediction, rather than downstream prognostication or administrative classification.

Data Extraction

A standardized data extraction framework was developed before synthesis. For each included study, information was recorded on authorship, publication year, clinical setting, study population size, data source, model type, use of real-time or continuous inputs, sepsis definition, prediction horizon, explainability features, and reported performance metrics. Additional information regarding internal validation, external validation, transfer learning, multicenter generalizability, and deployment context was extracted where available. This structured approach enabled comparison across studies with differing designs and model architectures.

Risk of Bias Assessment

Methodological quality and risk of bias were evaluated using the PROBAST framework [10], which examines the following four domains: participants, predictors, outcomes, and analysis. Particular attention was given to retrospective cohort design, outcome-label derivation, handling of missing data, overfitting risk, calibration reporting, sample size adequacy, and external validation methods. Studies were categorized as having low, moderate, or high overall risk of bias based on domain-level concerns and relevance to the review question.

Data Synthesis

Because of substantial heterogeneity in study populations, sepsis definitions, model architectures, prediction horizons, and reported metrics, a quantitative meta-analysis was not considered appropriate. Instead, findings were synthesized narratively with emphasis on patterns in predictive performance, interpretability approaches, transportability across institutions, and clinical implementation relevance. Particular attention was given to chronological trends from earlier score-based systems to contemporary DL models and externally validated deployment-oriented platforms.

Results

Study Selection Process

**Figure 1:**
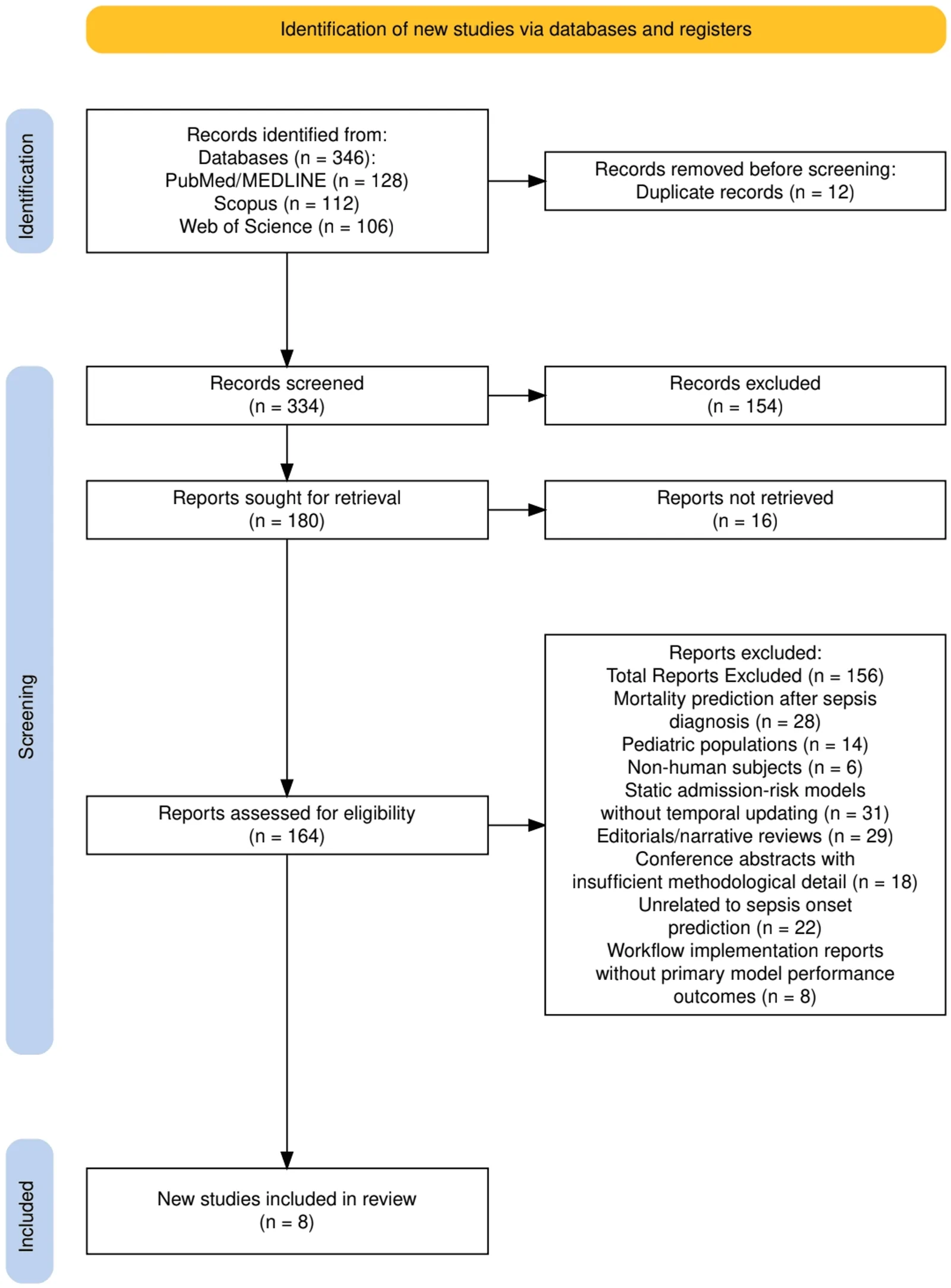
Preferred Reporting Items for Systematic Reviews and Meta-Analyses (PRISMA) flow diagram illustrating the study selection process for inclusion in the systematic review of real-time artificial intelligence models for early sepsis prediction.

As illustrated in Figure 1, the literature search identified 346 records across PubMed/MEDLINE, Scopus, and Web of Science. After removal of 12 duplicates, 334 records underwent title and abstract screening, of which 154 were excluded. A total of 180 reports were sought for retrieval, with 16 unavailable for full-text review. Subsequently, 164 full-text articles were assessed for eligibility, and 156 were excluded for predefined methodological or scope-related reasons. Ultimately, eight studies met the inclusion criteria and were included in the final qualitative synthesis.

Characteristics of the Selected Studies

As summarized in Table 1, the eight included studies evaluated real-time AI models across ICUs, EDs, and multicenter hospital systems [12-19]. Sample sizes ranged from relatively small validation cohorts to more than 500,000 patients, and the evaluated approaches included early warning scores, gradient-boosting models, recurrent neural networks, and other DL systems. Reported discrimination was generally moderate to high, with AUROC values ranging from approximately 0.76 in external validation to above 0.95 in some cohorts. Prediction windows varied considerably, from 1-5 hours before sepsis onset to a median of 28.2 hours before septic shock, while only a subset of studies incorporated explicit explainability methods or extensive external validation. Further study-level details are presented in Table 1.

**Table 1:** Characteristics and performance of real-time artificial intelligence models for early sepsis prediction using dynamic clinical data. AI: artificial intelligence; ICU: intensive care unit; ED: emergency department; EHR: electronic health record; AUROC: area under the receiver operating characteristic curve; AUPRC: area under the precision-recall curve; SHAP: Shapley additive explanations; LSTM: long short-term memory; CNN: convolutional neural network

Study	Setting	Population (n)	AI model	Real-time/Continuous Input	Sepsis definition	Prediction horizon	Explainability	Performance
Valan et al., 2025 [12]	Four EDs, community health system multisite external validation	205,005 encounters; 101,584 unique patients	Sepsis Watch (machine learning model)	Yes	Early sepsis detection cohort	Early detection stated; exact fixed lead time not specified in abstract	Not primary focus	AUROC = 0.906–0.960; AUPRC = 0.177–0.252
Moor et al., 2023 [13]	International multicenter ICUs (US, Netherlands, Switzerland)	136,478 ICU admissions	Deep learning system	Yes (hourly resolved monitoring)	Sepsis-3	Detected 80% cases 3.7 hours before onset	Not primary focus	Internal AUC = 0.846; external AUC = 0.761; transfer-tuned AUC = 0.807
Chen et al., 2022 [14]	ICU; MIMIC-III + Ruijin Hospital + real-world ICU validation	6,891 + 453 + 67 patients	LightGBM, MLP, ensemble with transfer learning	Yes	ICU sepsis prediction cohort	1–5 hours preceding onset	Yes (SHAP)	AUC = 0.94 on HDRJH; 0.86–0.90 real-world ICU validation
Shashikumar et al., 2021 [15]	Two US healthcare systems; ICU + ED; external and temporal validation across six cohorts	515,720 patients	COMPOSER (deep learning with conformal prediction)	Yes	Sepsis risk cohorts	ICU median 12.2 hours prior to antibiotics; ED 2.1 hours	Partial (indeterminate/reject-option logic; robustness-focused)	AUC ICU = 0.925–0.953; ED = 0.938–0.945
Shashikumar et al., 2021 [16]	Three healthcare systems; hospitalized patients	Not clearly stated	DeepAISE (recurrent neural survival model)	Yes	Sepsis event prediction cohort	Early prediction of time-to-septic events	Yes (interpretable temporal/risk factor representations)	AUC = 0.87–0.90; FAR = 0.20–0.25
Lauritsen et al., 2020 [17]	Multicenter Danish hospitals (beyond ICU), 7-year retrospective dataset	Not clearly stated	CNN + LSTM deep learning model	Yes (EHR event sequences)	Sepsis incidence detection cohort	AUROC at 3 hours and 24 hours before onset	No clear explainability component	AUROC = 0.856 (3 hours), 0.756 (24 hours)
Nemati et al., 2018 [18]	ICU; two Emory hospitals + MIMIC-III external validation	~27,000 development + ~42,000 validation after exclusions	Artificial Intelligence Sepsis Expert (AISE)	Yes (hourly vitals + EMR data)	Sepsis-3	4, 6, 8, 12 hours before onset	Yes (top contributing factors)	AUROC = 0.83–0.85
Henry et al., 2015 [19]	ICU patients	Not clearly stated	TREWScore (targeted real-time early warning score)	Yes (continuous EHR physiological + laboratory data)	Septic shock risk	Median 28.2 h before shock onset	Partial (score-based model)	AUC = 0.83; sensitivity 0.85 at specificity 0.67

Risk of Bias Assessment

As presented in Table 2, risk of bias was assessed using the PROBAST framework across the domains of participants, predictors, outcomes, and analysis. Overall, most included studies were judged to have a moderate risk of bias, largely reflecting the retrospective design of the underlying cohorts, variation in sepsis labeling methods, and incomplete reporting of calibration or missing-data handling. Studies with large multicenter external validations generally demonstrated lower concern in the participants domain, whereas smaller single-center transferability studies showed greater analytical uncertainty related to limited validation sample sizes and potential model instability. The analysis domain represented the most frequent source of concern across studies, particularly regarding overfitting risk, threshold selection, and limited prospective confirmation. No study was considered entirely free of bias concerns, although several recent investigations demonstrated stronger methodological rigor through temporal or external validation.

**Table 2:** Risk of bias assessment of prediction model studies using the PROBAST framework. PROBAST: Prediction model Risk Of Bias ASsessment Tool; AI: artificial intelligence; ICU: intensive care unit; EHR: electronic health record

Study	Tool used	Participants	Predictors	Outcome	Analysis	Overall risk of bias
Valan et al., 2025 [12]	PROBAST	Low	Low	Moderate	Moderate	Moderate
Moor et al., 2023 [13]	PROBAST	Low	Low	Moderate	Moderate	Moderate
Chen et al., 2022 [14]	PROBAST	Moderate	Low	Moderate	High	High
Shashikumar et al., 2021 [15]	PROBAST	Low	Low	Moderate	Low	Moderate
Shashikumar et al., 2021 [16]	PROBAST	Moderate	Low	Moderate	Moderate	Moderate
Lauritsen et al., 2020 [17]	PROBAST	Moderate	Low	Moderate	Moderate	Moderate
Nemati et al., 2018 [18]	PROBAST	Low	Low	Moderate	Moderate	Moderate
Henry et al., 2015 [19]	PROBAST	Moderate	Low	Moderate	Moderate	Moderate

Discussion

Principal Findings

The present review suggests that real-time AI for early sepsis prediction has evolved from proof-of-concept scoring systems to more sophisticated, clinically oriented decision-support platforms [12-19]. Across the included studies, most models demonstrated moderate-to-high discriminatory performance, with reported AUROC values ranging from approximately 0.83 in earlier studies to above 0.90 in several contemporary cohorts [12-19]. Prediction horizons varied substantially, ranging from alerts within a few hours of sepsis onset to identification windows exceeding 12 hours [13-19]; notably, Henry et al. reported a median lead time of 28.2 hours before septic shock [19]. More recent investigations increasingly emphasized false-alert mitigation, external validation, transferability, and deployment across heterogeneous care settings, reflecting a shift in priorities beyond model discrimination alone [12-16]. Nevertheless, the evidence base remained predominantly retrospective, limiting certainty regarding causal clinical benefit [12-19]. Collectively, these findings indicate that the field has progressed from demonstrating algorithmic prediction of sepsis toward determining whether these systems can be trusted, generalized, and effectively integrated into routine clinical care.

Predictive Performance Trends

A consistent pattern across the literature is the gradual improvement in reported discrimination metrics over time; however, these gains should be interpreted with caution. Earlier studies, such as those by Henry et al. [19] and Nemati et al. [18], reported AUROC values in the range of 0.83 to 0.85, whereas later studies, including those by Shashikumar et al. [16] and Valan et al. [12], reported AUROC values exceeding 0.90 in certain cohorts. However, AUROC alone provides an incomplete assessment of clinical utility, particularly in relatively low-prevalence conditions, where positive predictive value may remain modest despite strong discrimination. This limitation is highlighted by the inclusion of the AUPRC in studies such as Valan et al. [12], which offers a more informative measure in the setting of class imbalance. Furthermore, high AUROC values do not preclude excessive false alerts, an issue directly addressed by Shashikumar et al. [15] through the use of indeterminate outputs designed to reduce unnecessary alarms. Calibration was infrequently and inconsistently reported across studies, despite its central importance for threshold-based clinical decision-making. Importantly, prediction lead time may be more clinically meaningful than marginal improvements in AUROC; for example, a model with an AUROC of 0.88 that reliably identifies sepsis four hours earlier with acceptable specificity may be clinically superior to a model with an AUROC of 0.93 that generates late or noisy alerts. Future evaluations should therefore balance discrimination with calibration, alert burden, and actionable lead time to better reflect real-world clinical utility.

Compared with conventional scoring systems, AI-based sepsis prediction tools differ principally in how they process clinical information. Scores such as SIRS, qSOFA, and NEWS apply predefined thresholds to a relatively limited set of variables and generally provide a static or intermittently recalculated assessment that is straightforward for clinicians to interpret [4,5]. In contrast, AI models can continuously integrate longitudinal changes in vital signs, laboratory results, and EHR data, allowing them to recognize nonlinear patterns and evolving physiological trajectories before conventional thresholds are crossed [6,12-19]. The included AI studies generally reported moderate-to-high discrimination and, in several cases, clinically relevant prediction lead times [12-19]. However, these apparent advantages must be balanced against greater computational and data requirements, reduced interpretability, susceptibility to dataset shift, and the potential for false-alert burden. Moreover, because conventional scores and AI models were not evaluated under uniform populations, sepsis definitions, prediction windows, and alert thresholds, the available evidence does not establish consistent clinical superiority of AI over conventional scoring systems. Prospective head-to-head studies are therefore required to determine whether improved predictive performance translates into earlier treatment and better patient outcomes.

Explainability and Trustworthiness

Explainability in sepsis prediction should be understood as more than the presence or absence of SHAP plots or ranked features. A more useful conceptual distinction is to view explainability as operating across at least three levels. Level 1 is feature attribution, in which the model identifies variables associated with elevated risk, as seen in studies such as Chen et al. [14], which used SHAP, and Nemati et al. [18], which reported major contributing factors. Level 2 is temporal reasoning transparency, in which the clinician can understand why the risk changed over time and which evolving physiological patterns drove the alert. This dimension is more closely approximated by Shashikumar et al. in DeepAISE [16], where interpretable temporal representations were part of the model design, but it remains underdeveloped across the literature. Level 3 is actionability, in which the system not only explains risk but also helps clinicians determine what should be verified or prioritized next in the patient assessment. Very few studies truly reached this level. Even Shashikumar et al. in COMPOSER [15], while highly important for trustworthiness because it allowed the model to abstain when uncertain, were oriented more toward robustness than bedside interpretive guidance. Taken together, the literature suggests that most sepsis AI systems remain concentrated at Level 1, with relatively limited progress toward temporal and actionable explainability. This distinction is important because feature attribution alone may improve transparency, but it does not necessarily improve clinical trust, decision quality, or safe implementation [20].

Generalizability and External Validity

A major finding across the included studies is that strong internal performance does not guarantee stable performance when models are transported across institutions, countries, or care environments. This issue is particularly well illustrated by Moor et al. [13], whose model performed well on internal validation but showed lower performance on external validation across international ICU datasets, with improvement only after fine-tuning on target-site data. By contrast, Valan et al. [12] demonstrated that Sepsis Watch retained strong performance across multiple EDs in a new health system, suggesting that some models may be portable when underlying workflows, data structures, and implementation contexts are sufficiently aligned. These differences likely reflect variation in coding practices, measurement frequency, antibiotic timing, patient case mix, and local definitions of sepsis onset, all of which can alter both predictors and labels. An important implication is that transportability may be more consequential than model architecture itself. In practice, a simpler model that is well calibrated and locally adapted may outperform a more complex imported model that was developed under different clinical and data-generating conditions. For this reason, future work should place greater emphasis on external validation, recalibration, and site-specific implementation testing rather than assuming that architectural sophistication alone will ensure real-world success.

Clinical Implementation Gap

An important theme emerging from this review is that successful prediction does not automatically translate into clinical benefit. The practical value of a sepsis model depends not only on discrimination but also on how alerts are delivered, interpreted, and acted upon within real workflows. Questions such as who receives the alert, whether it is directed to nurses, physicians, or rapid response teams, what threshold prompts escalation, and whether the resulting alert burden is manageable are central to implementation but are addressed inconsistently in the literature. This is evident in deployment-oriented work related to TREWS [19] and COMPOSER [15], where clinical outcomes and workflow integration became as important as model performance itself. Studies associated with Shashikumar et al. and the broader COMPOSER program [15] indicate that reducing false alarms and handling uncertainty are essential if alerts are to remain actionable rather than disruptive. Similarly, outcome-focused evaluations of TREWS suggest [19] that benefit may depend heavily on timely clinician confirmation and downstream response rather than the alert alone. These observations support the view that the main bottleneck in the field may have shifted from algorithm development to workflow integration. In other words, the central challenge is no longer simply building a model that predicts sepsis early, but embedding that prediction within a clinical pathway that improves treatment timeliness without increasing unnecessary intervention or alert fatigue.

Methodological Limitations of Existing Literature

The current evidence base remains limited by several recurring methodological weaknesses that complicate interpretation and comparison. Most included studies were retrospective, which constrains causal inference and raises concerns regarding dataset-specific optimism, temporal leakage, and the extent to which predictors were truly available at the moment of prediction. Sepsis definitions also varied, ranging from Sepsis-3-based labeling to septic shock endpoints and institution-specific operational criteria, thereby affecting both incidence and reported model performance. Prediction windows were heterogeneous, spanning one to five hours in some studies and more than twelve hours in others, making direct comparison of clinical utility difficult. Calibration reporting was generally limited, despite its importance for threshold selection and bedside deployment, and prospective validation studies remained relatively sparse. In addition, subgroup fairness analyses were infrequently reported, leaving uncertainty regarding model performance across demographic and clinical subpopulations. Comparator strategies also varied considerably, with some studies benchmarking against conventional scores, others against ML baselines, and some providing limited comparative context. More broadly, the field suffers from benchmark fragmentation, making direct model comparison difficult. This fragmentation, combined with probable publication bias toward better-performing models, suggests that the apparent progress in sepsis AI should be interpreted with appropriate caution.

Future Research Agenda

Future progress in sepsis prediction will depend less on producing additional retrospective models and more on generating evidence that addresses clinical deployment, transparency, and measurable patient benefit. First, model evaluation should proceed through staged prospective validation, beginning with silent trials in which predictions are generated without influencing care, followed by pragmatic randomized or stepped-wedge trials that test real-world effectiveness under routine conditions. Second, reporting standards should be strengthened through consistent use of frameworks such as PROBAST [10] to improve reproducibility, transparency, and critical appraisal. Third, studies should routinely report calibration performance and decision-curve analyses alongside discrimination metrics, as accurate risk ranking alone does not ensure that threshold-based decisions are clinically appropriate. Fourth, explainability should move toward a human-centered model that helps clinicians understand changing risk over time, verify likely causes of deterioration, and prioritize next diagnostic or therapeutic steps rather than merely displaying feature rankings. Fifth, endpoints should increasingly reflect outcomes that matter to patients and health systems, including mortality, ICU-free days, timeliness of antibiotic administration, organ dysfunction trajectories, and alert burden. Advancing these priorities would shift the field from technical promise toward clinically accountable AI for sepsis care.

## Conclusions

The current literature indicates that real-time AI can identify patients at risk of sepsis earlier than conventional recognition pathways, with many contemporary models demonstrating strong discriminatory performance and clinically meaningful lead times. However, the most important challenge is no longer whether sepsis can be predicted, but whether these predictions can be delivered in a manner that is trustworthy, transferable, and capable of improving patient outcomes across diverse care settings. The evidence reviewed suggests that progress in model architecture has outpaced progress in prospective validation, calibration reporting, workflow integration, and demonstration of consistent clinical benefit. Future success will therefore depend on shifting emphasis from isolated performance metrics toward implementation-ready systems that combine accuracy with interpretability, external validity, and measurable reductions in morbidity and mortality. The central take-home message is that the next generation of sepsis AI should be judged not by how well it predicts sepsis in retrospective datasets, but by how reliably it improves care at the bedside.
